# First case of infant botulism in Sicily—case report

**DOI:** 10.1186/s13052-024-01798-4

**Published:** 2024-11-06

**Authors:** Antonino Fazzino, Carmelinda Cavallaro, Francesca Cavataio, Giulia Linares, Antonina Lo Cascio, Carla Lo Porto, Giuseppe Santangelo, Laura Venuti, Giovanni Corsello, Claudia Colomba

**Affiliations:** 1 Division of Pediatric Pulmonology, Children’s Hospital “G. Di Cristina”, ARNAS Civico-Di Cristina-Benfratelli, Via Dei Benedettini 1, Palermo, 90134 Italy; 2 Department of Health Promotion, Mother and Child Care, Internal Medicine and Medical Specialties “G. D’Alessandro”, University of Palermo, Palermo, Italy; 3 Division of Pediatric Gastroenterology, Children’s Hospital “G. Di Cristina”, ARNAS Civico-Di Cristina-Benfratelli, Via Dei Benedettini 1, Palermo, 90134 Italy; 4 Division of Pediatric Infectious Diseases, “G. Di Cristina” Hospital, ARNAS Civico Di Cristina Benfratelli, Via Dei Benedettini 1, Palermo, 90134 Italy; 5 Primary Care Pediatrician, Medical School of Pediatrics, University of Palermo, Palermo, Italy; 6 Division of Child Neuropsychiatry, “G. Di Cristina” Hospital, ARNAS Civico Di Cristina Benfratelli, Via Dei Benedettini 1, Palermo, 90134 Italy

**Keywords:** Infant botulism, Hypotonia, Botulinum toxin, Clostridium botulinum, Case report

## Abstract

**Background:**

Botulism is a rare and life-threatening disease caused by the potent botulinum neurotoxin (BoNT), which can be produced by Clostridium botulinum (*C. botulinum*) and related bacteria. Clinical manifestations, which include a symmetrical, descending muscular paralysis, generalized hypotonia, and potentially respiratory failure, are non-specific and diagnosis is challenging, especially when anamnesis does not reveal any typical risk factor, like honey consumption.

**Case Presentation:**

We present what is, to the best of our knowledge, the first documented case of infant botulism (IB) in Sicily and discuss its peculiarities and the challenges faced in the diagnostic-therapeutic process. The infant was exclusively breastfed and no history of consumption of possibly contaminated foods, like honey, was found. The signs observed at presentation included poor suction, hypotonia, and hyporeactivity. A detailed anamnesis motivated the suspicion of botulism, due to the occurrence of constipation and exposure to dust from home renovation works during the days before the onset of symptoms. The botulinum antitoxin was administered and the diagnosis was confirmed through fecal examination, detecting toxin-producing *C. botulinum*.

**Conclusion:**

IB should be considered in every infant with rapidly progressing hypotonia and a history of constipation. However rarely, transmission could occur through inhalation of dust particles containing the toxin, therefore it is important to explore all possible sources of exposure. In the case described, timely diagnosis and treatment determined the successful outcome, which highlights the importance of early intervention in managing IB.

## Background

Botulism is a rare and potentially fatal disease caused by the neurotoxin produced by *Clostridium botulinum* (*C. botulinum*). It manifests with symmetric cranial nerve palsy initially, followed by a descending, flaccid paralysis affecting muscles symmetrically, including respiratory muscles and potentially leading to respiratory failure [1–3].

This condition is widespread globally, with foodborne botulism being the predominant form in Europe [2, 4]. In 2021, European Center for Disease Prevention and Control (ECDC) collected 82 reports of botulism, with a general incidence of 0.02 cases per 100,000 people [5]. Interestingly, the highest incidence rate of Europe was observed in Italy, with 0.03 cases per 100,000 people [4].

Around 60% of cases of infant botulism (IB) are associated with honey consumption [2]. Infants are vulnerable to the intestinal colonization of *C. botulinum* because of the immaturity and changes of the microbiome typical of this age [6–8]. The condition has an earlier onset and is more dangerous in formula-fed infants, probably due to their dietary lack of immune factors normally contained in human milk, including IgAs, lactoferrin, and lysozyme [9]. Breastfed infants face a heightened risk during the weaning period, with the introduction of solid foods [4]. While the source of the spores remains unidentified in many cases, there have been occasional reports of IB associated with the consumption of contaminated milk or cereal powder, herbal infusions, and untreated water and *C. botulinum* spores have been found in these sources [2, 4, 6, 10–13].

Risk factors for botulism, other than those related to the microbiome and formula-feeding, include slow intestinal motility, birth by cesarean section, living in a windy area, dust exposure at home, Meckel’s diverticulum, intussusception, and colitis associated with *Clostridium difficile* [6, 14–16]. Conversely, being the firstborn and breastfeeding are protective factors [6, 9].

The first case of IB in Italy was diagnosed in 1984 [17]. Between 1986 and 2015, 36 cases of IB were documented out of a total 466 confirmed cases of botulism in Italy [5]. We report what is, to the best of our knowledge, the first case of IB observed in Sicily, and provide an overview of this infrequent condition.

## Patient Report

A 4-month-old, Caucasian infant presented to the emergency department with poor suction, hypotonia and hyporeactivity. The infant was born from unrelated parents at 38 weeks of gestation by caesarean section, had an adequate birth weight and a regular perinatal period. The newborn metabolic screening yielded negative results. He was exclusively breastfed and had not yet started weaning. His growth chart and his neuromotor development were appropriate. About one week before admission to the hospital, he was vaccinated against Rotavirus. Upon admission, he was moderately dehydrated, poorly responsive, with a feeble cry, and normal vital signs. Over the preceding three days, he experienced inappetence and poor suction, in the absence of fever, vomiting, or diarrhea.

His hematochemical exams showed normal inflammatory markers, glucose, creatine phosphokinase, ammonia and lactate levels. The blood culture was negative, and an abdominal ultrasound (US) excluded the possibility of intussusception encephalopathy.

**Fig. 1:**
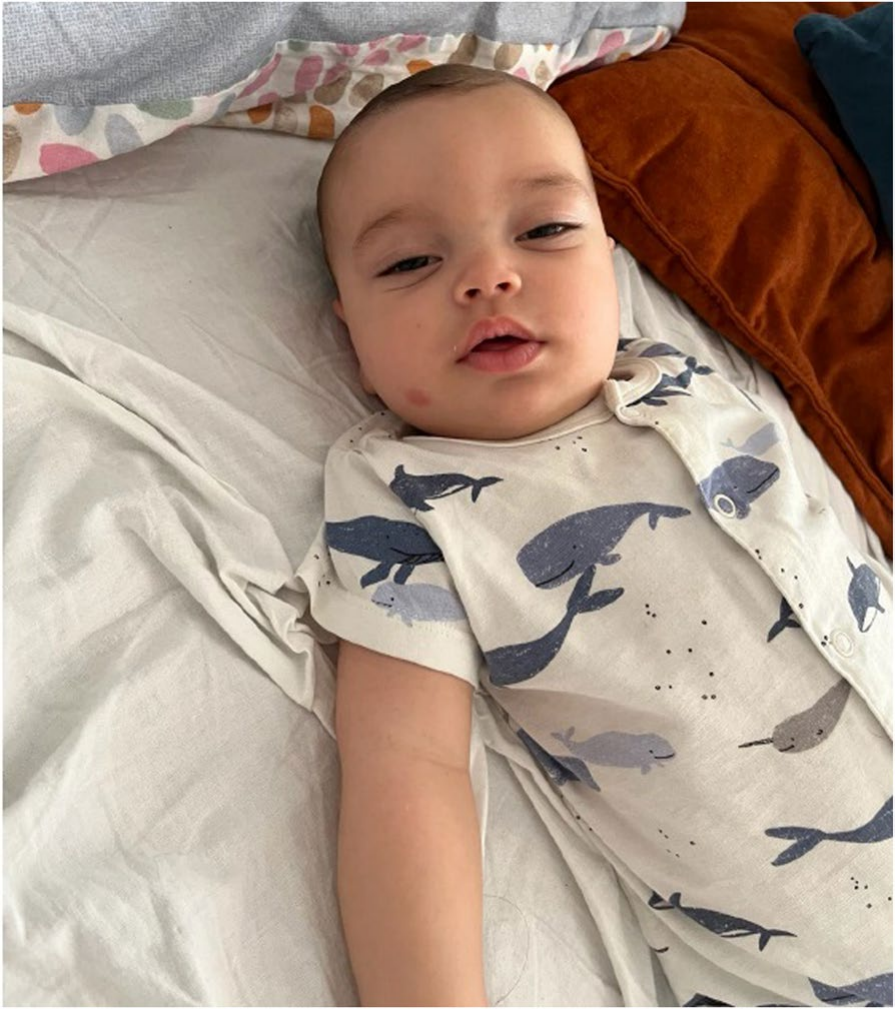
Ptosis, facial paresis with loss of facial expression in hypotonic infant

A more detailed neurological exam revealed poor suction, generalized axial hypotonia, reduced head control, marked weakness, reduced motor control of the limbs, a weak and hoarse cry, flattened facial expressivity, bilateral ptosis, and mydriasis (Fig. 1).

Accordingly, a toxicologic screening test was carried out and resulted negative. Brain imaging, including computed tomography (CT) and magnetic resonance imaging (MRI), did not show any cerebral injuries. The cerebrospinal fluid was sterile and the cell count was within the normal range. Similarly, the electroencephalography did not show any pathological sign.

Through a more thorough anamnestic investigation, a history of constipation starting 5 days prior to admission was found. Additionally, while denying that the infant consumed any honey, the parents revealed that home renovation works were ongoing at the grandmother’s house and the infant had frequently been exposed to construction dust. For this reason, in the suspicion of botulism, stool samples were obtained through an enema and submitted to the Italian reference center for botulism, the “Centro Nazionale di Riferimento per il Botulismo” (CNRB).

Thirty-six hours since admission, the patient developed respiratory failure and was transferred to the intensive care unit to be intubated. Considering the concrete clinical suspicion of IB, in accordance with the indications of the poison control center of Pavia (“Centro Antiveleni di Pavia”), we decided to administer the botulism antitoxin.

During the hospital stay, neuromuscular diseases and metabolic disorders associated with flaccid paralysis were excluded. On the fifth day since admission, the patient’s general condition improved and he was extubated.

The following day, the diagnosis of IB was confirmed, in light of the outcome of the fecal examination conducted at the CNRB, which revealed the presence of toxin-producing *C. botulinum* through polymerase-chain reaction (PCR).

**Fig. 2:**
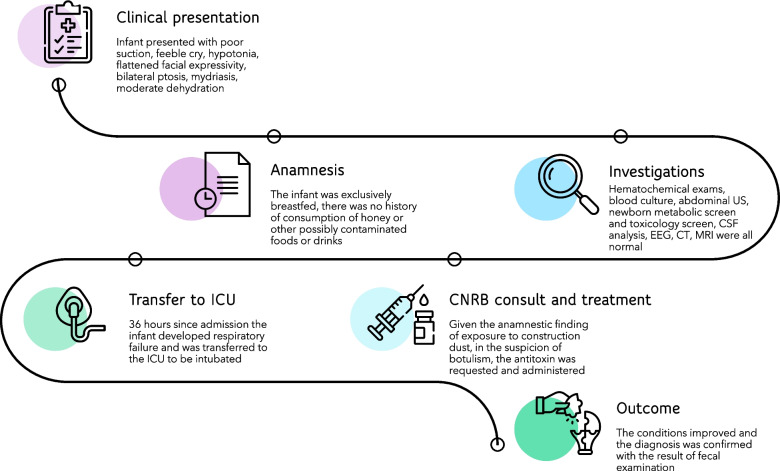
Illustration of a case of infant botulism from initial presentation to outcome (described in text)

The patient was discharged on the twentieth day. Upon discharge, he was able to feed spontaneously and, over the course of the following weeks, he continued neuromotor rehabilitation for a persistent mild, generalized hypotonia. Muscular tone restored completely within three months without any sequelae. An overview of the main steps of the diagnostic-therapeutic process is illustrated in Fig. 2.

## Discussion and Conclusions

### Etiopathogenesis

*C. botulinum* is an anaerobic, spore-forming, Gram-positive bacillus [2, 6]. Its spores are commonly present in soil, household dust, and agricultural products [8]. They are resistant to boiling and cooking and are often ingested by humans, however, they do not normally germinate in the intestine [1].

Botulinum toxins are produced by *C. botulinum* and related species and are considered the most powerful biological toxins known [1, 4]. There are 9 different types of BoNTs, but only 4 (A, B, E, and F) are associated with human botulism [2, 6]. BoNTs can reach the bloodstream in several ways. Based on the entering route of the toxin, different forms of botulism can be distinguished, such as foodborne botulism, iatrogenic botulism, wound botulism, and inhalational botulism, which is due to exposure to aerosolized BoNTs, possibly associated with occupational settings or bioterrorism [1, 3, 4, 6]. Significantly, the same type of *C. botulinum* that affected the patient has been discovered in household dust in instances of IB [9, 18, 19]. In the case we reported, botulism was likely acquired through inhalation of BoNT-laden dust particles generated through home renovation works. Dilena et al. reported a similar case of IB in a six-month-old infant, exclusively breastfed, whose father was a carpenter involved in home renovation [6].

Another form of botulism is intestinal toxemia, characterized as IB when it affects children under one year of age [6, 20, 21]. Unlike foodborne botulism, which results from the accidental ingestion of the preformed toxin, intestinal toxemia is due to spore germination, toxin production and colonization of Clostridia in the large intestine [2, 3, 6].

The synthesis of BoNTs includes several steps. The progenitor toxin releases a single-chain polypeptide in the upper small intestine, which, in turn, is cleaved in two subunits: a heavy chain (H-chain) and a light chain (L-chain) [8]. The H-chain binds to membrane glycoproteins of the presynaptic membrane at the neuromuscular junction, leading to the endocytosis of the L-chain, which blocks the release of acetylcholine in the synaptic space [1, 2, 8]. The outcome is therefore flaccid paralysis and extremity weakness [2]. Since the intestinal smooth muscles are the first to be affected, constipation is an early sign [2]. Head, facial and throat musculature is also affected early on for circulatory reasons, with subsequent bulbar palsy [8]. What follows is a descending flaccid paralysis of voluntary and autonomic muscles, potentially leading to respiratory failure [1–3].

### Clinical manifestations

Signs and symptoms of botulism can last from hours to a few days and occur after an incubation period spanning from 12–36 h to 10 days [1, 2, 8, 22].

The clinical picture can range from a mild syndrome, with poor feeding, dry mouth, constipation and drowsiness resolving over a few days, to severe hypotonia and respiratory failure [2, 8]. Three phases can be distinguished: (a) a descending paralysis lasting two weeks, (b) a phase of minimum muscle function of the same duration, and (c) a slow, lengthy muscle-recovery phase [6, 7]. Initial manifestations include ptosis, flat expressivity, weak cry and drooling; hypotonia, loss of head control and paralysis follow; respiratory paralysis can occur at last, with a mortality rate of 1% [1, 2]. Since mydriasis could also be a sign in infants, and it is not always promptly noticeable, it is advisable to repeat the pupillary test for two minutes [8].

Recovery occurs through regeneration of nerve axons, leading to motility restoration [2, 20]. In order to ward off the risk of aspiration, patients should be discharged only when they are gagging, sucking and swallowing effortlessly [2, 8]. Complications of IB include hypoxic brain injury, cardiac arrest, syndrome of inappropriate antidiuretic hormone secretion (SIADH), urinary tract infections due to indwelling bladder catheters, septicemia associated with intravascular catheters, and pneumonia [2, 8].

Both clostridia and toxins can be found in the feces of infants for weeks or months after symptoms resolution [2, 8].

### Diagnosis

Considering the rarity of botulism and the lack of clinical specificity, it is unsurprising that the diagnosis is often delayed or missed [1]. This condition should be considered in all hypotonic infants with feeding difficulties, ptosis and constipation [2, 6].

Diagnosis can be confirmed through the identification of *C. botulinum* or BoNTs in microbiological samples, such as serum or stools [2, 6]. Toxin identification takes about 48 h, while culture of Clostridia requires more than 5 days [2].

The Italian CNRB uses polymerase-chain reaction (PCR) together with the standard mouse bioassay method to confirm the diagnosis of botulism [17].

Possible differential diagnoses include drug intoxication, sepsis, which is the most frequent misdiagnoses upon admission, metabolic disorders, and neurologic conditions such as Guillain Barré Syndrome (GBS), encephalitis, meningitis, and spinal muscular atrophy (SMA) type 1 [1, 6, 8].

Blood tests, cerebrospinal fluid exam, and imaging investigations are non-specific but can be helpful to manage the complications and to exclude other diagnoses, such as sepsis, meningitis and dehydration [2]. Nerve conductions studies and electromyography can reinforce the clinical suspicion while waiting for the microbiological confirmation [2, 6].

### Treatment

Treatment consists of supportive care, including respiratory and nutritional support, and administration of the botulinum antitoxin, which decreases mortality and length of hospital stay [1, 20]. The antitoxin should be administered as soon as possible even if the diagnosis has not been confirmed and, in case of progressive paralysis, it should always be administered regardless of the time of symptom onset [1, 2]. If neurologic signs persist 24 h from the administration of antitoxin, alternative diagnoses should be considered [1]. While the antitoxin cannot reverse paralysis, it neutralizes toxins that are not yet bound to synaptic receptors [1].

Human intravenous botulism immunoglobulin (BIG-IV) is the gold standard treatment for IB, while the equine botulinum antitoxin (EqBA) can be considered as an alternative [2]. BIG-IV consists of IgGs able to neutralize type A and type B BoNTs [23]. Its recommended dosage is 50 mg/kg and it should be administered only once, intravenously [2]. On the other hand, EqBA is derived from horses hyperimmunized with BoNTs [2]. In 2010, a botulism antitoxin heptavalent (BAT) formulation neutralizing 7 BoNT serotypes was approved and licensed by the Food and Drug Administration (FDA) [1]. In Europe, the BAT and the trivalent botulinum type A + B + E antitoxin are the most commonly used for botulism forms other than IB [2]. The equine antitoxin is associated with the risk of anaphylaxis and lifelong sensitization to equine proteins; epinephrine and antihistamines should be readily available at the time of administration [1]. Practical details of the treatment with antitoxins are described in Table 1.

**Table 1:** Treatment of infant botulism: comparison between BAT and BIG-IV

	***Eptavalent/BAT***	***BIG -IV in IB***
Formulation	A 20 mL vial contains 7 antitoxins [2, 24]: - serotype A: 4500 UI - serotype B: 3300 UI - serotype C: 3000 UI - serotype D: 600 UI - serotype E: 5100 UI - serotype F: 3000 UI - serotype G: 600 UI	Lyophilized powder, stabilized with 1% albumin and 5% sucrose, with a 5% content of human IgG, in particular: - AB neutralizing toxin type A: 15 IU/mL; - AB neutralizing toxin type B: 4 IU/mL. [2, 25, 26]
Mechanism of action	Equine IgG Fab or F(ab’)2 fragments inactivate BoNTs type A to G, preventing its binding to the cholinergic nerve endings [2, 24]	Human IgG inactivate BoNTs type A and B [2, 25]
Half-life	5–8 days [2, 27]	- 28 days in infants; - Adequate blood concentration to neutralize all free BoNT that enters the infant’s circulation for at least the following 6 months. [2, 23, 25]5/5/2024 5:47:00 PM
Dose	- For adults the appropriate dose is 1 vial; - For infants, 10% of the adult dose, or 1/10 of a single vial; - For children aged 1–16 years, 20–100% of the adult dose, according to the Salisbury Rule: i. ≤ 30 kg: 2 × child’s weight (kg) ii. > 30 kg: child’s weight (kg) + 30. [2, 24]	50 mg/kg [1 ml/kg] intravenously, in a single dose [2, 25]
Administration	Should occur within 5 days since symptom onset, after checking for hypersensitivity* [2, 24, 27] In case of negative preliminary skin test, administer a 1:10 dilution in saline solution [2, 24] For children, the initial infusion rate should be 0.01 ml/kg/h, to be increased of 0.01 ml/kg/h per time, until a maximum rate of 0.03 ml/kg/h [24] For adults (17 years or older), an initial infusion rate of 0.5 mL/min can be doubled until a maximum rate of 2 mL/min [24]	- Initiate administration within 2 h from reconstitution and complete it within 4 h; - The initial infusion rate should be 0.5 ml/kg/h (25 mg/kg/h), which may be increased one time to 1.0 ml/kg/h (50 mg/kg/h), in the absence of adverse reactions; - Never exceed the indicated dose, concentration and infusion rate. [2, 25]
Warnings and Contraindications	Patients at increased risk of hypersensitivity, such as those who already received an equine-derived antitoxin, have allergies (including to horses) or asthma, should be monitored carefully [24] A risk of infectious disease transmission exists due to the use of equine plasma in the preparation [24]	Administration is contraindicated in case of history of severe reaction to other human IgG and selective IgA deficiency with anti-IgA ABs Consider the possibility of hypersensitivity reactions, hyperproteinemia, hyponatremia, thrombotic events (especially in risk groups), hemolytic anemia, pulmonary adverse reactions, infectious diseases. [25]
Adverse reactions	- Headache, nausea, pruritus, and urticaria observed in ≥ 5% of healthy volunteers; - Pyrexia, rash, chills, nausea, and edema were reported in ≥ 1% of patients in a clinical study; - Hemodynamic instability occurred in one patient in the clinical study. [24]	The most frequent side effect (5% of children in a trial) was a mild erythema of the face or trunk. [25]

Characteristics and practical indications for treatment with botulinum antitoxin. Extended from Antonucci et al. [2]

Acronyms: *BAT *Botulism antitoxin heptavalent, *BIG-IV* Human intravenous botulism immunoglobulin, *AB* Antibody, *IB* Infant botulism

^*^The hypersensitivity check is performed by injecting in the volar surface of the forearm 0.2 mL of EqBA diluted 1:1000 (0.9% saline solution) and comparing to histamine control after 15–20 min [2]

Mechanic ventilation should be considered when swallowing and gagging are compromised in order to prevent respiratory arrest and hypoxic encephalopathy [1, 8]. Nutritional support through a nasogastric tube promotes peristalsis and the elimination of Clostridia [8].

Thanks to modern intensive care techniques, mortality rates have been decreasing over the past decades and patients who receive adequate supportive care can recover completely even without the administration of the antitoxin [1]. Of note, Nevas et al. reported a case of IB characterized by its presentation with sudden infant death syndrome (SIDS) [28]. The infant passed at 11 weeks and *C. botulinum* was found in his intestinal content and in dust collected from his household [28].

Antibiotic treatment is not recommended for IB for several reasons: (1) lysis of bacteria could increase the amount of free toxin in the colon, (2) the intestinal flora may be altered in a way that facilitates the growth of Clostridia, and (3) aminoglycosides may enhance neuromuscular block [1, 3, 8, 29].

### Conclusion

The reported case is, to the best of our knowledge, the first case of IB observed in Sicily. The subtle clinical presentation observed in this case demonstrates the importance of clinical suspicion in diagnosing IB. It is advisable to consider this condition in every infant with rapidly progressing hypotonia and a history of constipation.

The peculiarity of this case lies in the mode of transmission of the disease, which likely consisted of toxin inhalation. Considering that honey consumption had been ruled out during anamnesis, botulism originally seemed an unlikely occurrence. However, a more thorough and careful consideration of all the potential sources of BoNTs led to the correct diagnosis. Our experience highlights the importance of taking a detailed medical history when IB is suspected, inquiring not only on honey consumption, but also on the possible exposure to construction work dusts or contaminated water or soil. Physicians should be aware that infants could accidentally inhale toxins from domestic or soil dusts or carried by relatives who work in building sites.

Furthermore, our case shows how a timely diagnosis signifies the administration of life-saving treatment.

## Data Availability

All clinical data and materials are available in Division of Pediatric Infectious Diseases, “G. Di Cristina” Hospital, ARNAS Civico Di Cristina Benfratelli, Via dei Benedettini 1, 90,134 Palermo, Italy.

## Abbreviations

BAT: Botulism antitoxin heptavalent
BIG-IV: Human intravenous botulism immunoglobulin
BoNT: Botulinum neurotoxin
CNRB: “Centro Nazionale di Riferimento per il Botulismo”
CT: Computed tomography
EqBA: Equine botulinum antitoxin
ECDC: European Center for Disease Prevention and Control
FDA: Food and Drug Administration
IB: Infant botulism
MRI: Magnetic resonance imaging
PCR: Polymerase-chain reaction
SIADH: Syndrome of inappropriate antidiuretic hormone secretion
SMA: Spinal muscular atrophy
US: Ultrasound
